# Analysis and control of fungal deterioration on the surface of pottery figurines unearthed from the tombs of the Western Han Dynasty

**DOI:** 10.3389/fmicb.2022.956774

**Published:** 2022-08-15

**Authors:** Yu Wang, Cen Wang, Xinyu Yang, Kaixuan Ma, Peifeng Guo, Qirui Sun, Shili Jia, Jiao Pan

**Affiliations:** ^1^Ministry of Education Key Laboratory of Molecular Microbiology and Technology, Department of Microbiology, College of Life Sciences, Nankai University, Tianjin, China; ^2^Institute of Cultural Relics and Archaeology, Jinan, China; ^3^Department of Cultural Relics and Museums, College of History and Culture, Qufu Normal University, Qufu, China; ^4^Institute of Cultural Heritage and History of Science and Technology, University of Science and Technology Beijing, Beijing, China

**Keywords:** tombs of the Western Han Dynasty, pottery figurines, fungal deterioration, fungistatic experiment, cultural heritage protection

## Abstract

In April 2020, 232 tombs of the Western Han Dynasty were found in Sundayuan, Heze City. In total, 141 pottery figurines of significant historical, cultural, and artistic value were unearthed from the tombs. Some of the figurines are currently being stored in warehouses, and the surface of some of the figurines show fungal deterioration. To thoroughly analyze the fungal deterioration on the surface of the pottery figurines and find appropriate control methods, we used high-through sequencing, scanning electron microscopy observation, pure cultures of culturable fungi, and optical microscopy observation and molecular identification of culturable fungi. We conducted fungistatic and simulation experiments in the laboratory to find appropriate control methods. We found that the fungi on the surface of the figurines were mainly of the phylum Ascomycota, and a few fungi were of the phyla Basidiomycota and Mucoromycota. We isolated seven culturable fungal strains and observed their colony morphology. The seven fungal strains were *Lecanicillium aphanocladii*, *Penicillium aurantiogriseum*, *Clonostachys rosea*, *Mortierella* sp., *Mortierella alpina*, *Aspergillus flavus*, and *Cladosporium halotolerans*. Through the fungistatic and simulation experiments conducted in the laboratory, we found that 50 mg/ml cinnamaldehyde and 0.5% K100 (2-methyl-4-isothiazolin-3-one) have a good fungistatic effect. They can not only inhibit the growth of fungi on medium, but also inhibit the growth of fungi on the surface of pottery figurines. This study has good reference significance for the analysis and control of fungal deterioration of unearthed pottery figurines.

## Introduction

An increasing number of studies have been focusing on the biological weathering and degradation of cultural relics, including monuments, murals, art exhibits, historical manuscripts, archaeological sites, and tombs. These studies show that cultural relics can provide ideal habitats for microbial colonization and growth (Pruvost et al., 2011; Sterflinger and Pinzari, 2012; Sterflinger and Piñar, 2013; Piñar et al., 2015). Fungi are ubiquitous colonists, and according to some researchers, they are the most important factor in the deterioration of cultural heritage materials in the outdoor environment (Masaphy et al., 2014; Liu et al., 2022). Over time, cultural relics are often contaminated and colonized by fungi (Milanesi et al., 2006; Pepe et al., 2010). Therefore, biological degradation caused by fungi has been considered an important aspect of cultural relics protection in the past 40 years (Sampo and Mosca, 1989). Many cultural relics contain organic substances that support fungal growth. It is well known that fungi can accelerate the degradation of cultural relics physically and chemically through mycelium growth and metabolites, respectively (Imperi et al., 2007; Sterflinger and Pinzari, 2012). Studies have reported that fungal hyphae can penetrate and expand cracks in rocks and building materials and produce a wide range of enzymes and other compounds, including pigments, leading to erosion, discoloration, and peeling of stones and murals (Sterflinger, 2010; Unković et al., 2016; Li et al., 2019).

From April 2020 to July 2021, the Shandong Institute of Cultural Relics and Archaeology and the local cultural relics department of Heze City explored and excavated the Sundayuan site. It was discovered that the whole site contains 232 ancient tombs. An analysis of the shape and objects of the tombs showed that these brick coffin tombs are most likely from between the middle and late periods of the Western Han Dynasty (from 202 B.C. to 8 A.D.). During the excavation, 141 exquisitely painted pottery figurines were found buried in brick coffin tombs. A significant number of the pottery figurines unearthed from the Sundayuan site are stored in warehouses. Owing to the high temperature and humidity of the storage environment, fungal deterioration has appeared on the surface of some of the pottery figurines.

The methods of controlling cultural relic diseases are mainly divided into the following categories: physical, chemical, and biological methods. Physical methods involve using physical technologies to inhibit the microorganisms causing cultural relic diseases. For example, ultraviolet rays can be used to damage microorganisms, destroy their nucleic acid function, and thus inactivate them to inhibit microorganisms on cultural relics (Scheerer et al., 2009). Titanium dioxide is a kind of nanoparticle that can produce peroxides and other components that can inhibit the microorganisms causing cultural relic diseases. This method has fewer side effects. Currently, this technology is being used in the protection of a few stone cultural relics (Russa et al., 2012; Coutinho et al., 2016). Although this method has been applied to the protection of cultural relics, further investigation is needed into whether it has the potential to cause harm to cultural relic workers. Certain mechanical methods are also included among physical methods. Mechanical methods refer to the method of manually removing organisms from the surface of cultural relics by directly using scalpels, tweezers, vacuum cleaners, and other tools. This method is simple to implement and has a significant inhibitory effect on the diseases of cultural relics caused by algae, lichens, and mosses. However, the disadvantage of using this method is that it cannot remove fungi that have invaded the interior of cultural relics and cannot completely inhibit the growth of organisms (Municchia et al., 2018). Moreover, since sharp tools are used, operating them carelessly may cause damage to cultural relics. Chemical methods mainly refer to the use of various liquid antibacterial agents or the use of gas for fumigation. Although many kinds of chemical bacteriostatic agents are available on the market, the types of bacteriostatic agents that can be applied to cultural relics protection are very few. Currently, antibacterial agents that can be used in cultural relics protection mainly include formaldehyde polymers, quaternary amines, isothiazolinone, etc. (Polo et al., 2010). In addition to these liquid bacteriostatic agents mentioned above, some gases, such as formaldehyde and ethylene oxide, are often used for fumigation in the process of cultural relics protection, but these gases are very toxic and cause harm to cultural relic workers. Biological methods mainly refer to the inhibition of microorganisms on artifacts using metabolic substances produced by organisms. In recent years, scientists have been paying attention to the antibacterial and insecticidal properties of natural substances. Scholars collected the essential oils of different plants such as anise, clove, and laurel and tested them for their inhibitory effects on various microorganisms related to the degradation of heritage artifacts, such as *Aspergillus*, *Penicillium,* and *Fusarium* as well as *Bacillus,* and found that the inhibitory effect was good and targeted, and that the constituents of these essential oils included terpenes, aromatic aldehydes, terpene aldehydes, and phenolic compounds (Borrego et al., 2012; Ashraf et al., 2014). Marjanlo reported the high inhibitory activity of cumin oil against *Botrytis cinerea* and attributed this effect to the cumin aldehyde component in the oil (Marjanlo et al., 2009). Besides plant essential oils, it has been found that some secondary metabolites of microorganisms are also potential natural bacteriostatic agents, which are also easily synthesized in the laboratory because of their simple chemical structure. These secondary metabolites mainly include aliphatic, cyclic aliphatic, aromatic, and terpene components (Gazzano et al., 2013). For example, Silva showed that lipopeptide secondary metabolites produced by *Bacillus* have antifungal activity (Silva et al., 2017). Overall, these natural products with bacteriostatic activity have great application prospects in artifacts protection, as they are naturally non-contaminated, cause little harm to the environment and human body, and thus can be used directly as biocides, as well as serve as a basic model for the development of natural biocides.

Pottery was not only a tool in daily life, but also an object of cultural identification for ancient communities. Technological choices and manufacturing techniques demonstrate the level of understanding and evolution of specific cultural and social backgrounds (Tite, 2008; Peña and McCallum, 2009; De Bonis et al., 2013; Dal Sasso et al., 2014; De Vito et al., 2014; Giannossa et al., 2014). The research and protection of underground historical relics is of great significance. Underground historical relics and their burial places (such as caves, necropolises, monuments, catacombs, tombs, and crypts) are valuable heritage of human civilization (Koh and Birney, 2019; Botticelli et al., 2020; Luo et al., 2021). To analyze and study the fungal deterioration on the surface of the pottery figurines and determine the appropriate control measures for them, we visited the warehouse the pottery figurines were stored in for on-site observation in July 2021 and took some samples for analysis. This study took the unearthed pottery figurines as the research object, comprehensively analyzed the fungal deterioration on the surface of the figurines using various biological technologies, determined the suitable fungistatic products among the fungistatic agents currently used in the field of cultural relics protection, and actively tried a few environmentally friendly biological metabolic products as fungistatic agents. This study is of great significance for the storage and protection of the pottery figurines unearthed in the Sundayuan site, and it has reference significance for the protection of pottery figurines unearthed in other areas as well.

## Materials and methods

### Study site and sampled artifacts

The Sundayuan site is located in the southwest of Weilou reservoir in Heze City, Shandong Province, about 200 meters southwest of the original Sundayuan village (115°18′0.51″ E, 35°17′41.58″ N; Supplementary Figure 1). The site has a north–south orientation and a shuttle shape. It is approximately 220 meters long from north to south and 80 meters wide from east to west, with a total area of about 12,000 square meters. Four unearthed pottery figurines were selected for this study. Their cultural relic numbers are TN61E65M47: 2, TN61E65M47: 3, TN61E65M47: 5, and TN61E65M169: 1 (Supplementary Figure 2).

### SEM and optical microscope observation

**Figure 1:**
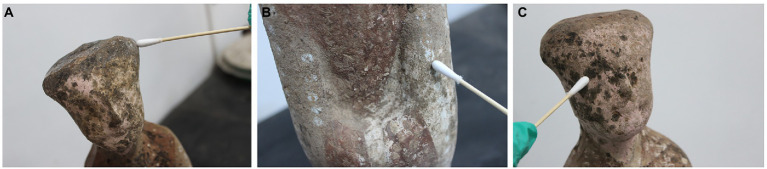
Spots on the surface of pottery figurines. **(A)** Ear of pottery figurine TN61E65M47:3. **(B)** Left arm of pottery figurine TN61E65M47:3. **(C)** Face of pottery figurine TN61E65M47: 2.

Three pottery figurines (cultural relic numbers TN61E65M47: 3, TN61E65M47: 2, and TN61E65M47: 5) were selected. Their surface had six spots suspected to be microbial colonization (Figure 1). The samples obtained using double-sided carbon conductive adhesive were glued to the sample cups and sprayed with gold. The gold-plated samples were observed with SEM (FEI Quanta 200, United States), and images were obtained at a magnification of 1,000× to 2,500×. An optical microscope (Nikon E200, Japan) was used to observe the morphology of mycelia and spores of dominant fungi. The microbial morphology was recorded under 40 times microscope.

### Total DNA extractions and high-throughput sequencing

Five pottery figurine samples (PF1–PF5) were selected for high-throughput sequencing. The five samples were taken from pottery figurines TN61E65M47: 5, TN61E65M47: 3, TN61E65M169: 1, and TN61E65M47: 2. DNeasy PowerSoil Kit (QIAGEN, Germany) was used to extract the genome DNA of all the samples. The total DNA was sent to Majorbio Genome Sequencing Company. The 18S rRNA gene region was sequenced using HiSeq platform. The primers used are SSU0817F’ (5′-TTAGCATGGAATAATRRAATAGG’-3′) and 1196R’ (5′-TCTGGACCTGGTGAGTTTC’-3′). The amplification regions are V5–V7 and the length of amplified fragment is 379 bp. The microbial community in the samples was analyzed using the sequencing results.

### Alpha diversity analysis

Alpha diversity was applied in analyzing the complexity of species diversity for a sample through five indices, including Sobs, Chao, Shannon, Simpson, and Good’s coverage. Sobs and Chao can reflect community richness, Shannon and Simpson can reflect community diversity, and Good’s coverage can reflect community coverage. All these indices in our samples were calculated using QIIME (Version 1.7.0) and displayed with R software (Version 2.15.3).

### Isolation and identification of culturable fungi

PDA medium was used to culture and isolate fungi. The PDA mediums were cultured at 28°C for 3–7 days. Fungi with significantly different morphology in each medium were isolated and purified. The T5 Direct PCR Mix kit (TSINGKE, China) was used for DNA extraction and PCR amplification of the dominant fungi according to the manufacturer’s protocol. The ITS1-5.8srRNA-ITS2 gene of the fungus was amplified with ITS1/ITS4 primers. The PCR system and conditions are detailed in the manual of the kit. The purified PCR products were sent to GENEWIZ (Beijing, China) for sequencing and then the sequence homology of the amplified fragments was analyzed by NCBI.

### Fungistatic experiment

The fungistatic efficacy of six biocides against the fungi isolated from the surface of the pottery figurines was examined. The biocides selected in our experiment can be divided into two categories: the biocides currently used for the protection of the cultural relics (K100, boric acid/borax solution, and miconazole nitrate) and the metabolites of plants and microorganisms (cinnamaldehyde, Glucosinolate crude extracts, and 5,5-Dimethyl-1, 3-cyclohexanedione). The concentration of various biocides was determined according to the literature and some previous experimental results. The fungistatic effects of 0.5% K100, 5% boric acid/borax solution (1: 1), and 0.5% miconazole nitrate were tested with ultrapure water as negative control, and the fungistatic effects of 50 mg/ml cinnamaldehyde, 2.7 mg/ml Glucosinolate crude extracts, and 0.5 g/ml 5,5-Dimethyl-1,3-cyclohexanedione were tested with methanol as negative control. The details of the biocides are provided in Supplementary Table 1. The experimental strains were fungi isolated from the surface of the pottery figurines. First, the strain was cultured by scribing on the PDA medium with a cotton swab, and the filter papers (each with a diameter of 7 mm) were placed on the medium at four places. One of the filter papers had the negative control, while the remaining three were impregnated with the different biocides (15 μl each). They were then placed in a 28°C incubator for culture. The fungistatic results were observed on the third day of culture.

### Laboratory simulation experiment

The fungus used in the experiment is *Lecanicillium aphanocladii* (NK-PF1). We selected 12 unearthed fragments of pottery figurines (Supplementary Table 2) as experimental materials, and added 1.5 ml well-distributed fungal suspension to them. Each fragment was then placed in a plastic box (27.5 cm × 20 cm × 16.5 cm) covered with gauze. The original condition of the pottery figurine fragments can be seen in Supplementary Figure 3. Subsequently, they were randomly divided into four groups. Each group was gently sprayed with 5 ml solvent of cinnamaldehyde (mixture of 1% dimethyl sulfate and 0.1% Tween-80), 50 mg/ml cinnamaldehyde, ultrapure water, and 0.5% K100 every day. They were cultured at room temperature (Average temperature 23°C) for 6 days. The growth of microorganisms on the pottery figurines was observed.

## Results

### SEM results of surface samples of pottery figurines

**Figure 2:**
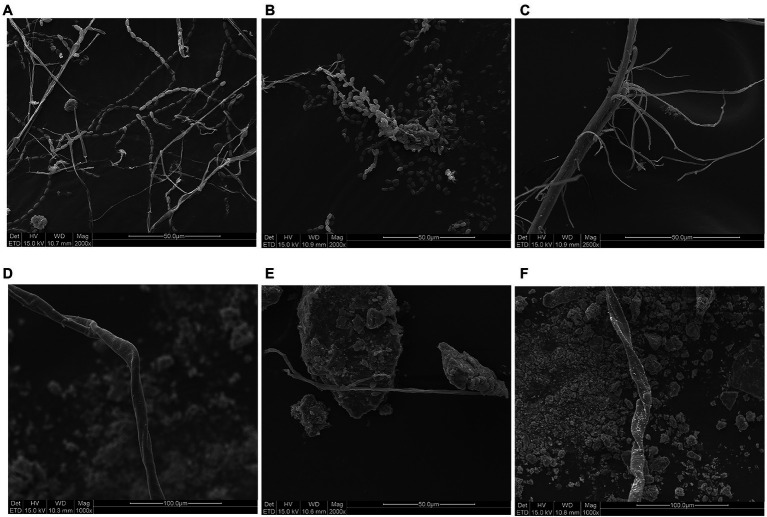
SEM results of spots. **(A)** Ear of pottery figurine TN61E65M47:3. **(B)** Face of pottery figurine TN61E65M47:3. **(C)** Left arm of pottery figurine TN61E65M47:3. **(D)** Face of pottery figurine TN61E65M47: 2. **(E)** Leg of pottery figurine TN61E65M47: 2. **(F)** Leg of pottery figurine TN61E65M47: 5.

SEM observation showed a large number of microorganisms on the ear and face of TN61E65M47:3. The mycelium and spores of fungi could be clearly seen at a magnification of 2000× (Figures 2A,B). There were no obvious microorganisms in the other four places (Figures 2C–F); these spots may have been caused by the peeling of the pigments on the surface of the pottery figurines or some chemical reactions.

### Fungal community analysis by high-throughput sequencing

**Figure 3:**
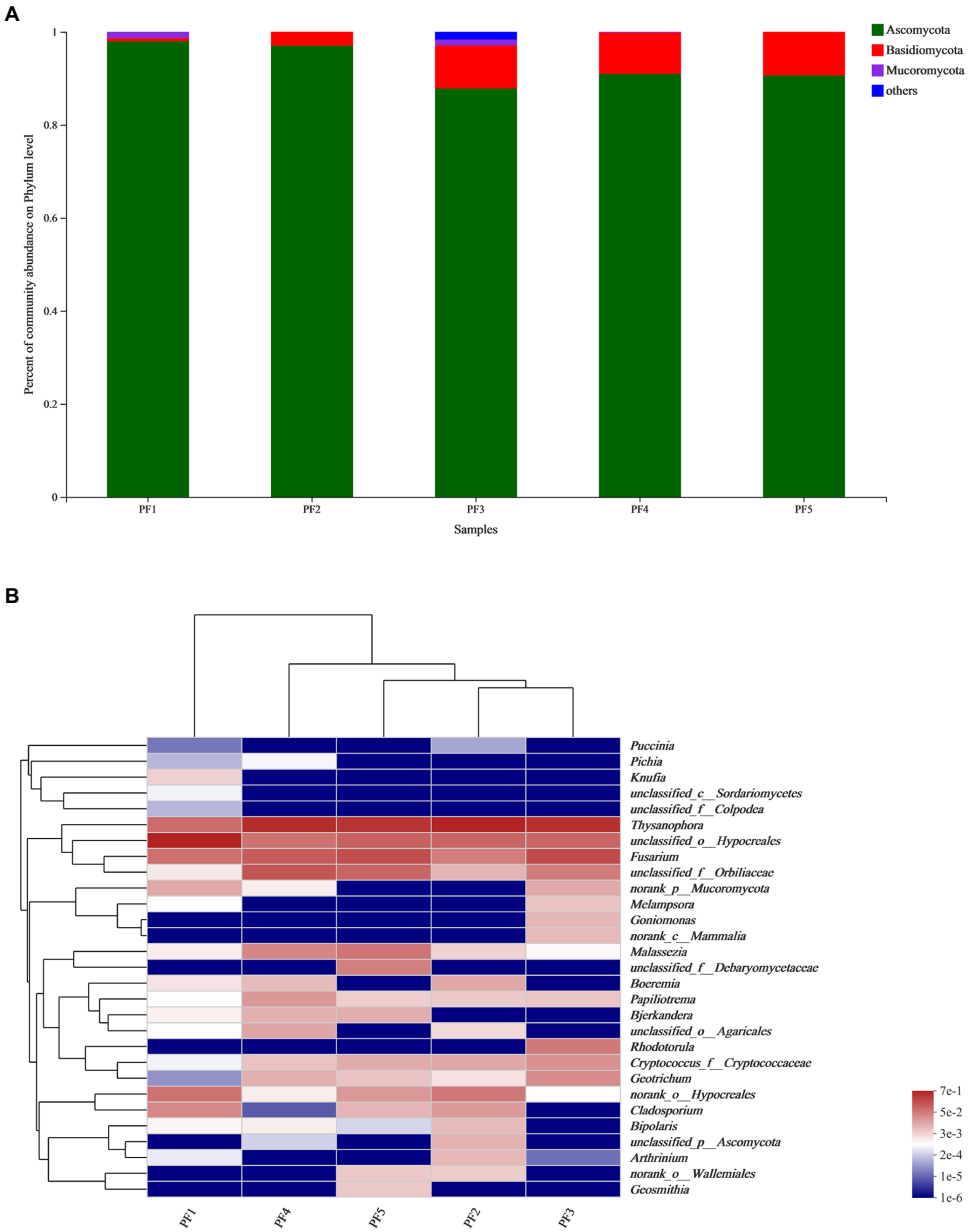
Fungal community analysis. **(A)** Histogram of species relative abundance of the pottery figurines at the phylum level; **(B)** Heatmap at the genus level.

It was found that most of the fungi on the surface of the pottery figurines were of the phylum Ascomycota, and a few were of the phyla Basidiomycota and Mucoromycota. The average proportion of Ascomycota reached 92.86%, while Mucoromyceta only existed in PF1, PF3, and PF4 (Figure 3A). In these 5 samples, *Thysanophora*, Hypocreales, *Fusarium*, and Orbiliaceae were present at higher levels and *Puccinia*, *Pichia*, *Knufia*, Sordariomycetes, and Colpodea were present at lower levels (Figure 3B). We simultaneously calculated the Alpha diversity index of these five samples. It can be seen that PF5 has the highest community richness and diversity, while PF1 has the lowest community richness and diversity (Table 1).

**Table 1:** Alpha diversity index.

Sample\estimators	Sobs	Shannon	Simpson	Chao	Good-coverage
PF1	24	1.13196	0.524766	24	0.99997
PF2	19	1.275372	0.504223	19	1
PF3	17	1.734827	0.283118	17	0.999975
PF4	18	1.618646	0.324113	18	0.999986
PF5	16	1.807785	0.238128	16	1

### Molecular identification and observation of purified fungi on the surface of the pottery figurines

**Figure 4:**
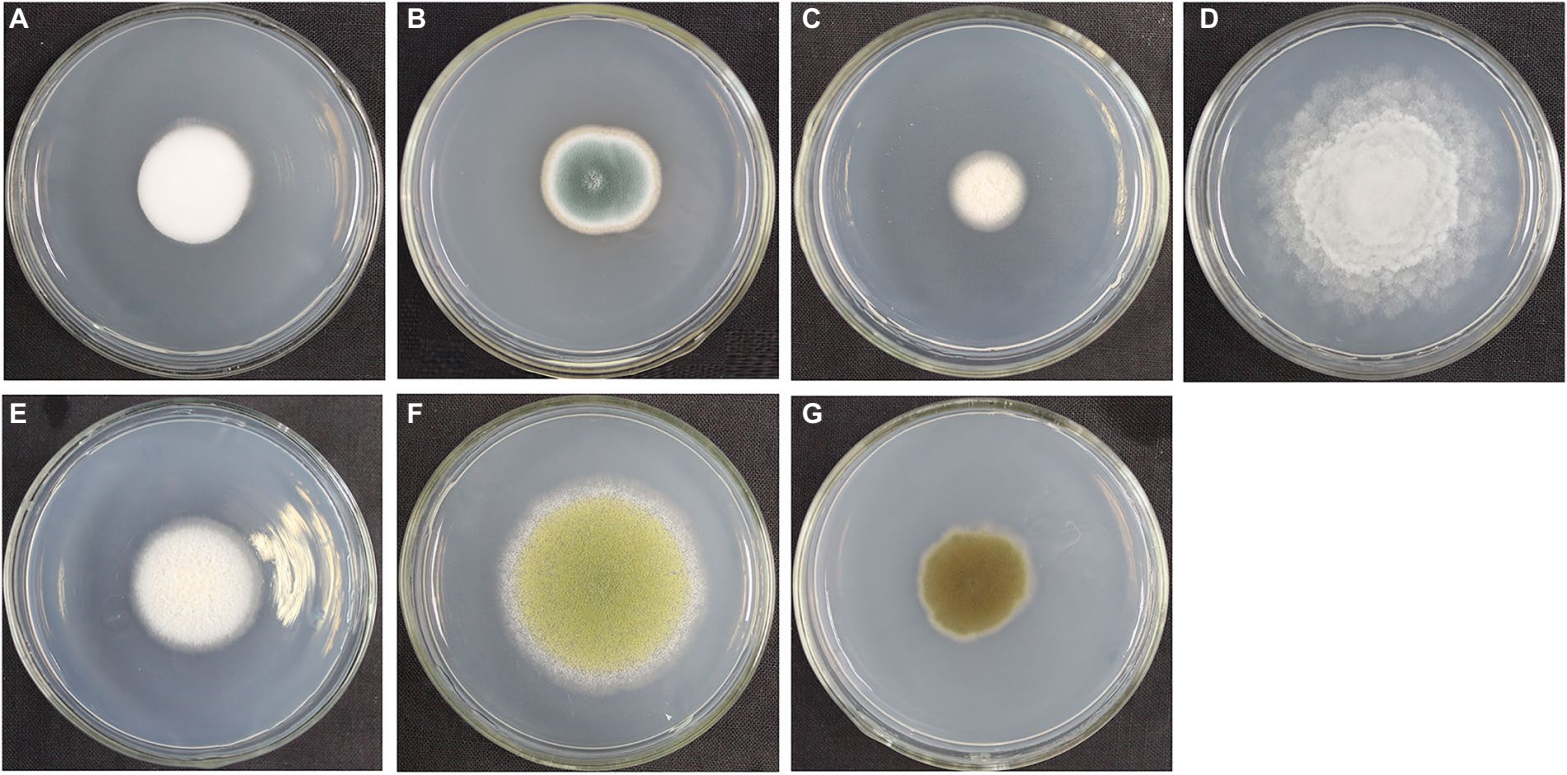
Single colony morphology of fungi isolated from the surface of pottery figurines. The fungi were inoculated onto the PDA medium and incubated at 28°C for 3 days. **(A)** *Lecanicillium aphanocladii* (NK-PF1). **(B)** *Penicillium aurantiogriseum* (NK-PF2). **(C)** *Clonostachys rosea* (NK-PF3). **(D)** *Mortierella* sp. (NK-I). **(E)** *Mortierella alpina* (NK-PF5). **(F)** *Aspergillus flavus* (NK-PF6). **(G)** *Cladosporium halotolerans* (NK-PF7).

**Figure 5:**
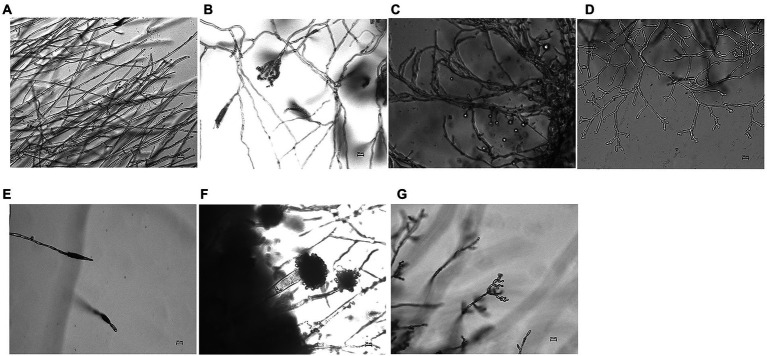
Micromorphology of fungi isolated from the surface of pottery figurines. The fungi were inoculated onto the PDA medium and incubated at 28°C for 3 days. The scale is 10 μm. **(A)** *L. aphanocladii* (NK-PF1). **(B)** *P. aurantiogriseum* (NK-PF2). **(C)** *C. rosea* (NK-PF3). **(D)** *Mortierella* sp. (IPF4). **(E)** *M. alpina* (NK-PF5). **(F)** *A. flavus* (NK-PF6). **(G)** *C. halotolerans* (NK-PF7).

We cultured seven fungal strains on PDA medium, namely, *L. aphanocladii*, *Penicillium aurantiogriseum*, *Clonostachys rosea*, *Mortierella* sp., *Mortierella alpina*, *Aspergillus flavus*, and *Cladosporium halotolerans* (Table 2). We observed their single colony state and observed their hyphae and spores using chamber culture and microscopy (Figures 4, 5).

**Table 2:** Molecular identification of strains isolated from the surface of the pottery figurines.

Fungi	Molecular identification results	GenBank accession no.
NK-PF1	*Lecanicillium aphanocladii*	ON945538
NK-PF2	*Penicillium aurantiogriseum*	ON945539
NK-PF3	*Clonostachys rosea*	ON945540
NK-PF4	*Mortierella* sp.	ON945541
NK-PF5	*Mortierella alpina*	ON945542
NK-PF6	*Aspergillus flavus*	ON945543
NK-PF7	*Cladosporium halotolerans*	ON945544

### Susceptibility of fungi to biocides

**Figure 6:**
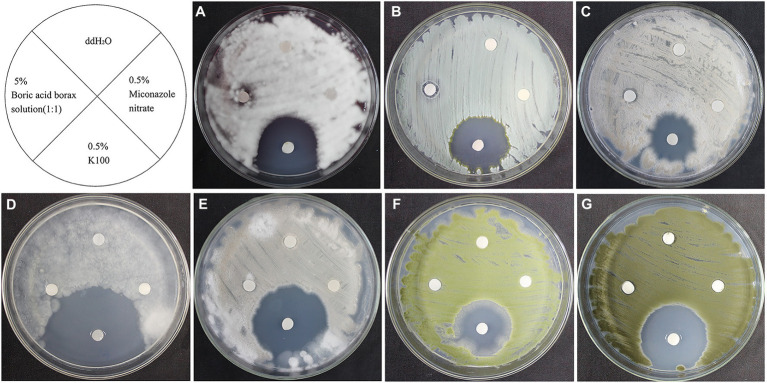
The fungistatic effects of 0.5% K100, 5% boric acid/borax solution (1:1), and 0.5% miconazole nitrate. The fungi were inoculated onto the PDA medium and incubated at 28°C for 3 days. **(A)** *L. aphanocladii* (NK-PF1). **(B)** *P. aurantiogriseum* (NK-PF2). **(C)** *C. rosea* (NK-PF3). **(D)** *Mortierella* sp. (NK-PF4). **(E)** *M. alpina* (NK-PF5). **(F)** *A. flavus* (NK-PF6). **(G)** *C. halotolerans* (NK-PF7).

**Figure 7:**
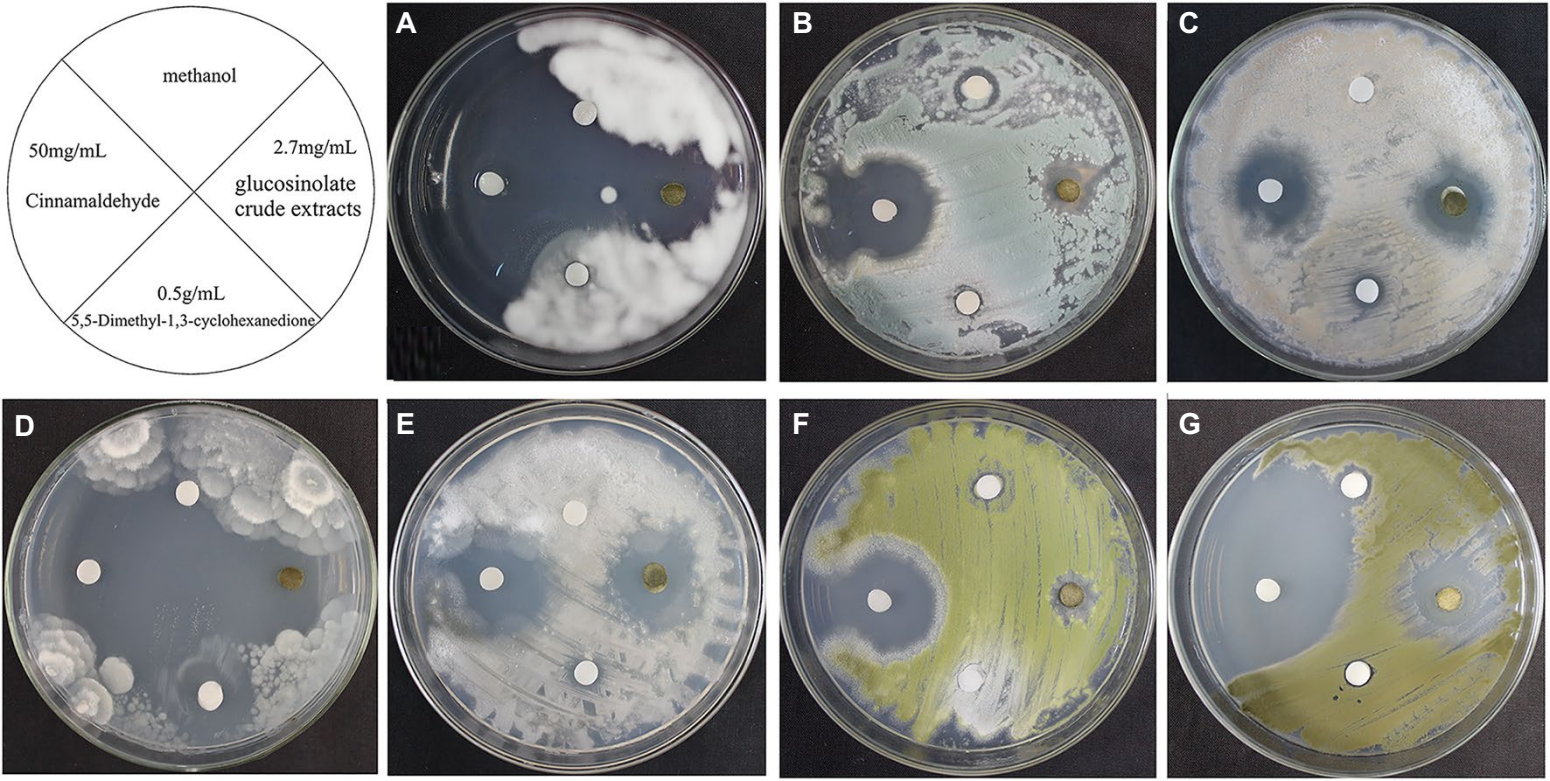
The fungistatic effects of 50 mg/ml Cinnamaldehyde, 2.7 mg/ml Glucosinolate crude extracts, and 0.5 g/ml 5,5-Dimethyl-1,3-cyclohexanedione. The fungi were inoculated onto the PDA medium and incubated at 28°C for 3 days. **(A)** *L. aphanocladii* (NK-PF1). **(B)** *P. aurantiogriseum* (NK-PF2). **(C)** *C. rosea* (NK-PF3). **(D)** *Mortierella* sp. (NK-PF4). **(E)** *M. alpina* (NK-PF5). **(F)** *A. flavus* (NK-PF6). **(G)** *C. halotolerans* (NK-PF7).

As shown in Figure 6, 0.5% miconazole nitrate showed no inhibitory effect on these 7 fungal strains and 5% boric acid/borax solution (1:1) showed only a slight inhibitory effect on *L. aphanocladii* (NK-PF1), *P. aurantiogriseum* (NK-PF2), and *C. rosea* (NK-PF3). 0.5% K100 showed a good inhibitory effect on all the fungi. As shown in Figure 7, 50 mg/ml cinnamaldehyde and 2.7 mg/ml glucosinolate crude extracts have certain inhibitory effects on these 7 fungal strains, and 50 mg/ml cinnamaldehyde has a better inhibitory effect than 2.7 mg/ml glucosinolate crude extracts. 0.5 g/ml 5,5-Dimethyl-1,3-cyclohexanedione also had a certain inhibitory effect on *Mortierella* sp. (NK-PF4).

### Simulation experiment results

**Figure 8:**
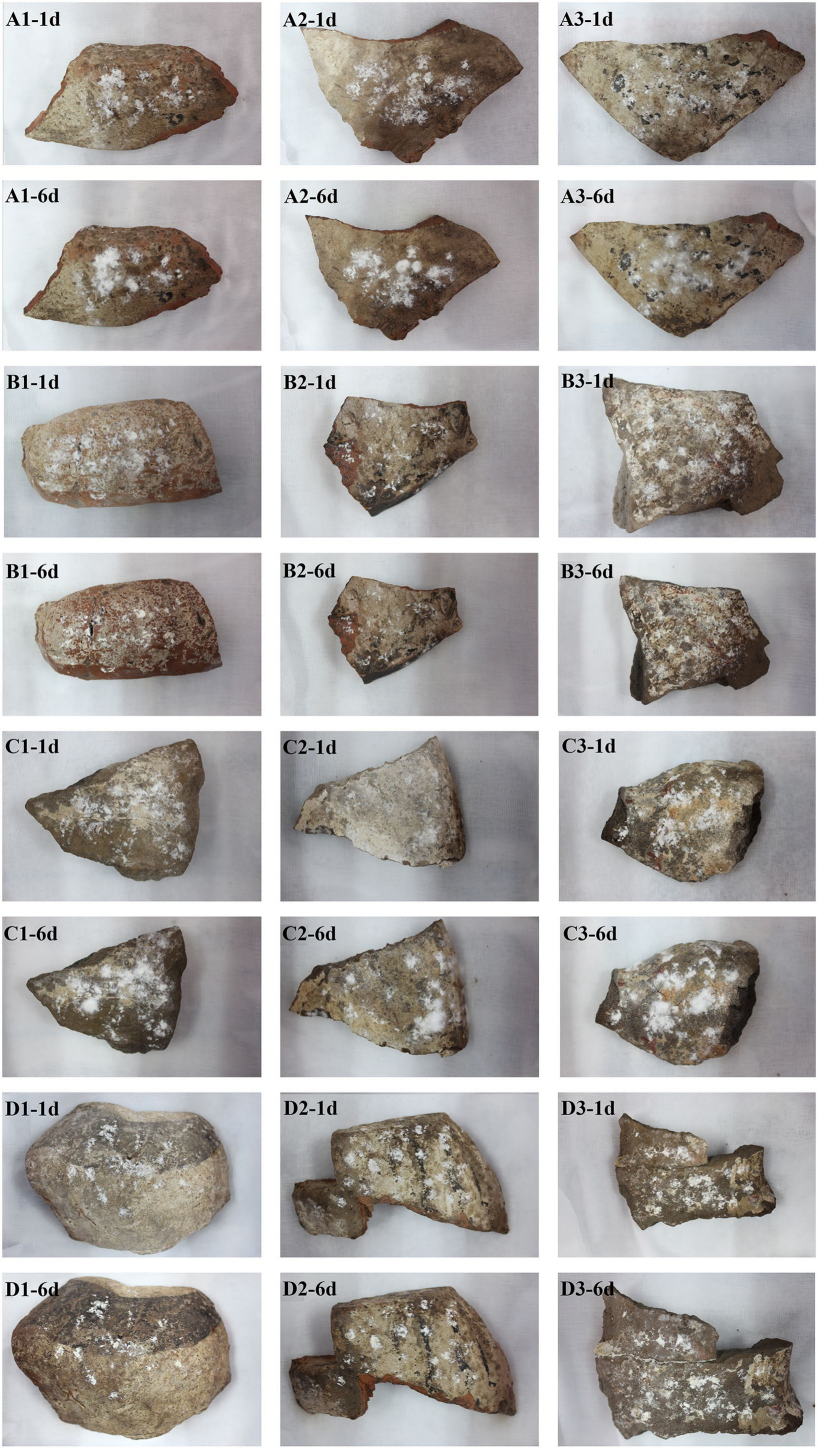
Pictures of pottery figurine fragments on day 1 (−1d) and day 6 (−6d) of the simulation experiment. Each group has three samples, and the same amount of NK-PF1 was added to each pottery figurine fragment. **(A)** Sprayed with 5 ml solvent of cinnamaldehyde (mixture of 1% dimethyl sulfate and 0.1% Tween-80) every day. **(B)** Sprayed with 5 ml 50 mg/ml cinnamaldehyde every day. **(C)** Sprayed with 5 ml ddH_2_O every day. **(D)** Sprayed with 5 ml 0.5% K100 every day.

Through the fungistatic experiment, we found that 0.5% K100 and 50 mg/ml cinnamaldehyde had better fungistatic effect. Therefore, we conducted the simulation experiment on controlling the growth of fungus on the surface of the pottery figurines in the laboratory. As shown in Figure 8, the fungi inoculated on the surface of pottery figurine fragments sprayed with ddH_2_O and a solvent of cinnamaldehyde could colonize and grow in large quantities, while the fungi on the surface of pottery figurine fragments sprayed with 0.5% K100 and 50 mg/ml cinnamaldehyde did not increase, indicating that spraying 0.5% K100 or 50 mg/ml cinnamaldehyde can effectively inhibit the growth and reproduction of disease fungi on the surface of pottery figurines.

## Discussion

In this study, fungal deteriorations on the surface of pottery figurines were thoroughly analyzed and studied. Using a scanning electron microscope, we found that not all the spots on the surface of pottery figurines were caused by fungal colonization. Some spots may have been formed by the chipping of the surface materials of the pottery figurines. As we can see in Figure 1, the surface of the pottery figurines were not smooth, the shedding of the material was visible, and the locations where the black spots were exactly concave were confirmed. These phenomena, together with the SEM observations, confirm our conjecture. Similar studies have been previously reported in the literature. After thousands of years of burial, the unearthed cultural relics have adapted to their burial environment. Even after centuries of burial, many historical relics are still well preserved (Cacace et al., 2003; Saiz-Jimenez et al., 2011). It is generally believed that the outstanding preservation of unearthed historical relics is due to the abnormal stability of internal climate parameters in the underground environment (Elez et al., 2013; Bourges et al., 2014). The thermal and humidity conditions in the burial environment are considered the basic factors for the integrity of historical relics. If cultural relics are excavated suddenly, violent fluctuations in temperature and relative humidity lead to irreversible deformation, such as curls and cracks. At the beginning of excavation, the Terra Cotta Warriors were intact and painted in vivid colors. Unfortunately, the raw lacquer underneath suffered severe warping, curling, and peeling, and the gray layer was exposed just a few hours after excavation (Zhang et al., 2007). Studies have also shown that some spots are evidence of microorganisms colonizing the surface. When studying the brown spots on the walls of the tomb of King Tutankhamun, Vasanthakumar et al. (2013) found through a scanning electron microscope that the primitive organisms that caused these spots no longer existed. Malic acid was detected only in the brown spots, not in the gypsum, indicating that microorganisms were involved in the formation of the spots. Many microorganisms, including fungi such as *Aspergillus* spp. (Peleg et al., 1988) and bacteria such as *Arthrobacter* and *Pseudomonas* (Vyas and Gulati, 2009) can produce malic acid in the environment.

The fungi on the surface of the pottery figurines may have come from the environment in which they were stored. The culturable fungi that colonize on the surface of cultural relics are closely related to their environment, and these fungi are potentially harmful to cultural relics (Samson et al., 2014). We isolated *Penicillium*, *Aspergillus*, and *Cladosporium* from the pottery figurines, and they are very common fungi, which are often isolated from the surface of cultural relics. For example, the predominant fungi in the air of Tianjin Museum are *Penicillium* and *Cladosporium* (Zhang et al., 2019a). *Cladosporium* was found in the air of an art museum in Tokyo (Abe, 2010). *Cladosporium* and *Penicillium* were also found in the ambient air test conducted for the storage of the Nanhai No. 1 shipwreck in 2019 and 2020 (Han et al., 2021), in the ambient air test for the Mogao Grottoes in Dunhuang from 2008 to 2009 (Wang et al., 2010a,b), and in the ambient air test for the canoe of the Tang Dynasty preserved in the National Marine Museum of China in 2019 (Zhang et al., 2019a). *Aspergillus* is one of the most abundant and widely distributed organisms on earth. It can produce a large number of conidia to spread widely (Pangallo et al., 2012). In addition, *Aspergillus* has the ability to produce a variety of enzymes and can colonize on any substrate. *Penicillium* and *Aspergillus* caused black spots in two 1700-year-old tombs (Ma et al., 2020). *Mortierella* has strong acid production capacity, and the organic acids produced by metabolism also have a deteriorative impact on cultural relics (Zhang et al., 2019b; IvančićŠantek et al., 2021). The hypha of *L. aphanocladii* is white. When growing on PDA medium, a red pigment is produced, which is a threat to cultural relics. This phenomenon has been previously reported (Dong et al., 2016). Therefore, controlling the storage environment is very important for the prevention and control of fungal deterioration. Controlling temperature and relative humidity is a key factor in cultural relics protection (Brimblecombe, 2013; Smedemark et al., 2020). In an indoor environment, fungi can grow with relatively low water content. Water accumulation promotes the growth of microorganisms on cultural relics, and humidity is positively correlated with fungal richness (Li et al., 2020; Trovão et al., 2020). When the temperature and humidity conditions are appropriate, fungal spores have the ability of long-term survival and growth (Vukojević and Grbić, 2010; Kakakhel et al., 2019). When we took samples, the temperature of the warehouse where the pottery figurines were stored was 26°C and the relative humidity was 70%. Such temperature and humidity conditions are conducive to the growth of fungi. Moreover, studies have reported that unsuitable temperature and relative humidity lead to mechanical, biological, and chemical degradation of cultural relics (Pinzari et al., 2020; Sadlowska-Salega and Radon, 2020). Currently, heating, ventilation, and air conditioning systems are the main methods used to meet the requirements of heat and humidity (Ferdyn-Grygierek and Grygierek, 2019; Schito et al., 2020). However, even if the excavated cultural relics can be stored in an environment with relatively stable temperature and humidity, secondary damage is still inevitable. Therefore, it is of great significance to study the environment before the excavation of cultural relics. It can serve as an important basis for preventive protection to avoid and minimize decay (Lucchi, 2018). We want to inhibit the fungi that grow on the surface of the pottery figurines using some targeted biocides. K100 and cinnamaldehyde were experimentally found to have good effects. The main component of fungistatic agent K100, which showed good fungistatic effect in this experiment, is a type of isothiazolinone. It can not only inhibit the growth of a variety of fungi on the culture medium, but also effectively control the growth and reproduction of the fungus *L. aphanocladiiz* on the surface of pottery figurines. Currently, K100 is also used as an effective fungistatic agent in the protection of the Nanhai No. 1 shipwreck (Han et al., 2021). However, as mentioned before, K100 may also have a slight impact on operators and the environment. Therefore, we are also looking for safer and environmentally friendly fungistatic substances. We also selected cinnamaldehyde, a plant essential oil, and crude glucosinolate extract from plants as fungistatic agents. Cinnamaldehyde is an aldehyde organic compound, which is a yellow, sticky liquid and abundantly found in plants such as cinnamon. It has a bacteriostatic effect and it can be added to aquatic products, meat products, etc., as a natural food preservative, so cinnamaldehyde is less harmful to humans and is promising for the protection of artifacts. In this experiment, fungistatic agents with a good inhibitory effect were selected. In the future, the abovementioned fungistatic agents can be considered for use on the pottery figurines to inhibit the fungal deteriorations on their surface.

## Conclusion

In this paper, the fungal deteriorations on the surface of pottery figurines unearthed from the Sundayuan site and their control were studied comprehensively. The results show that not all the spots on the surface of the pottery figurines were caused by fungal colonization. Moreover, the fungi that colonize on the surface of cultural relics are closely related to their environment. Two fungistatic agents, namely, K100 and cinnamaldehyde, can effectively inhibit the growth and reproduction of microorganisms on the surface of the pottery figurines.

## Data availability statement

The datasets presented in this study can be found in online repositories. The names of the repository/repositories and accession number(s) can be found at: https://www.ncbi.nlm.nih.gov/, PRJNA827628.

## Author contributions

JP conceived and designed the study, reviewed and edited the manuscript. YW, CW, and XY performed the experiments. KM and PG provided assistance during the experiments. QS and SJ provided samples. YW analyzed the data and wrote the manuscript. All authors contributed to the article and approved the final version of this manuscript.

## Funding

This work was continuously supported by the Natural Science Foundation of Tianjin (19JCZDJC33700).

## Conflict of interest

The authors declare that the research was conducted in the absence of any commercial or financial relationships that could be construed as a potential conflict of interest.

## Publisher’s note

All claims expressed in this article are solely those of the authors and do not necessarily represent those of their affiliated organizations, or those of the publisher, the editors and the reviewers. Any product that may be evaluated in this article, or claim that may be made by its manufacturer, is not guaranteed or endorsed by the publisher.
