# Supplementary material for: Analysis and control of fungal deterioration on the surface of pottery figurines unearthed from the tombs of the Western Han Dynasty
Source: Front Microbiol. 2022 Aug 15;13:956774. doi: 10.3389/fmicb.2022.956774 (PMC9421245; doi:10.3389/fmicb.2022.956774)
Supplement: Supplementary file 1 [file Data_Sheet_1.docx]

Supplementary Material


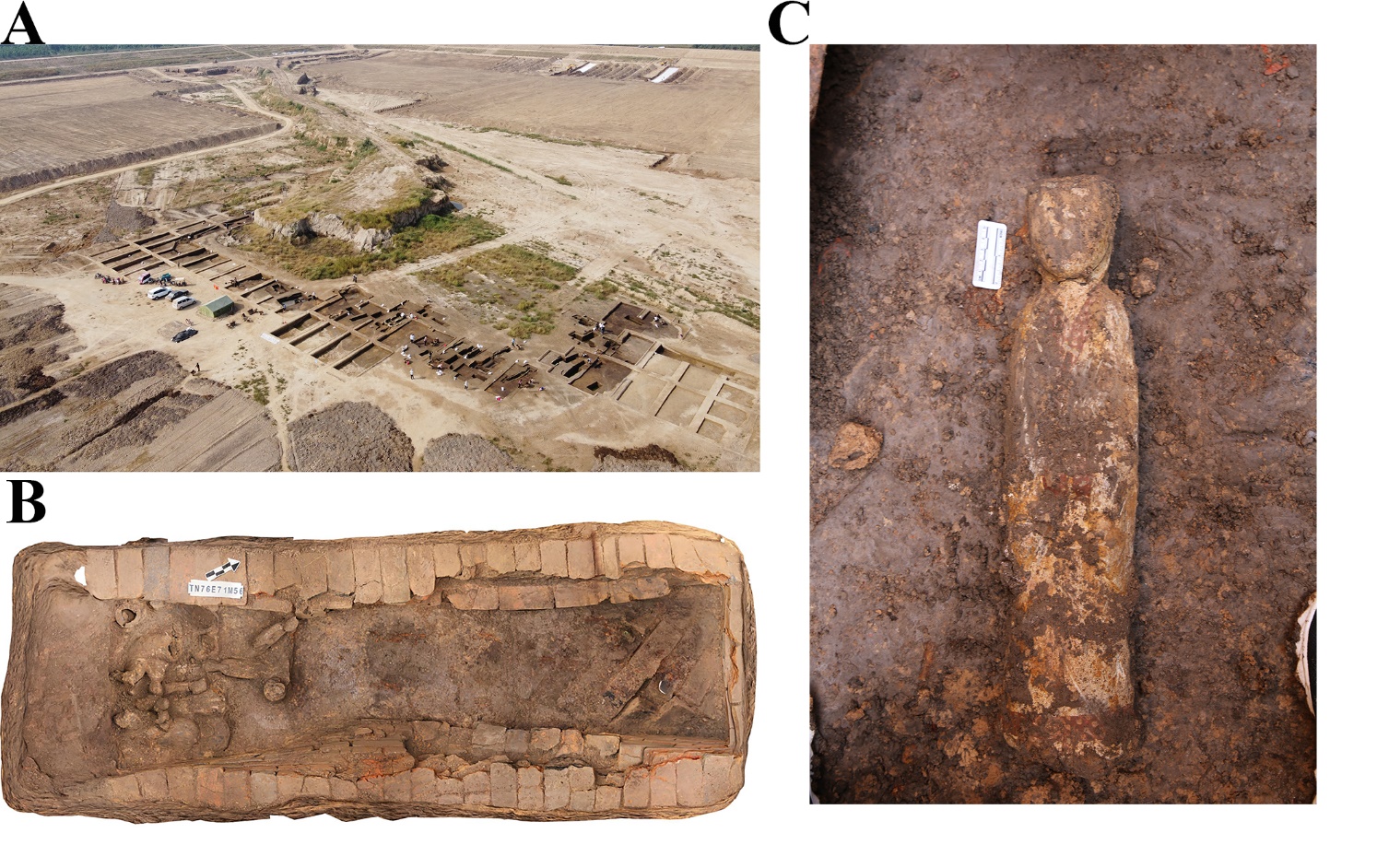


**Supplementary Figure 1**. Tomb excavation pictures. (A) Excavation site. (B) Tomb. (C) Pottery figurines in tombs.


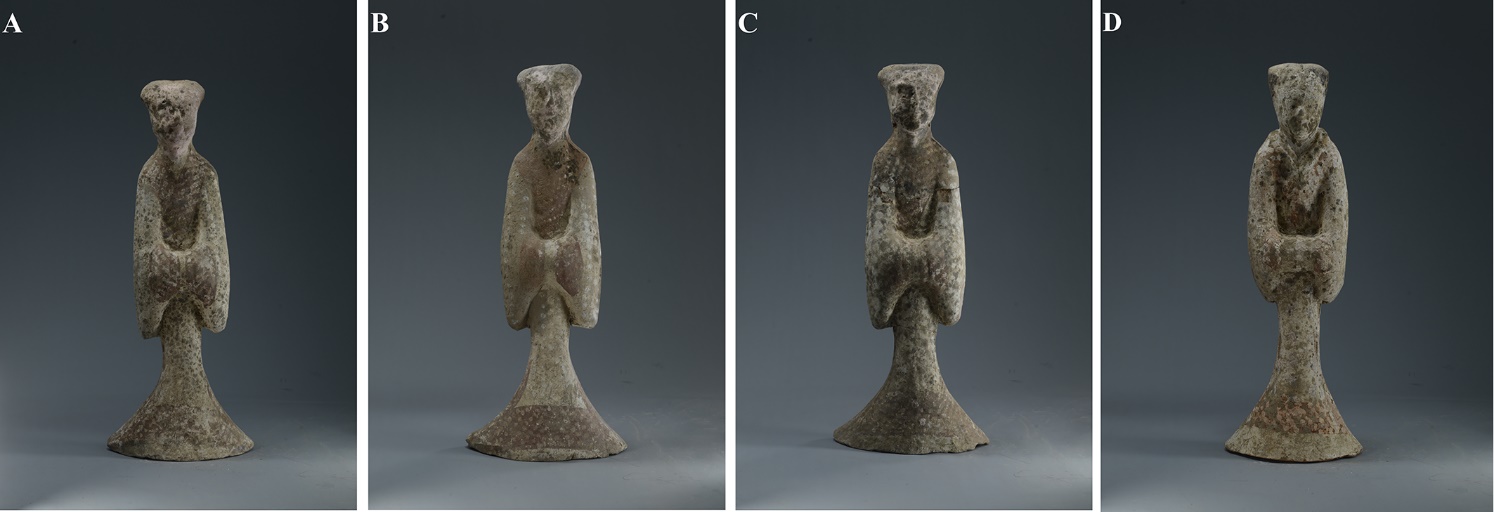


**Supplementary Figure 2**. Pottery figurines used in the experiment. (A) TN61E65M47:2. (B) TN61E65M47:3. (C) TN61E65M47:5. (D) TN61E65M169:1.

**Supplementary Table 1**. Information of fungistatic agents.

| Biocides | Molecular Formula | CAS Number | Solvent | pH | Suppliers |
| --- | --- | --- | --- | --- | --- |
| K100 (2-methyl-4-isothiazolin-3-one) | C_4_H_5_NOS | 2682-20-4 | ultrapure water | 6 | Guangzhou Tingkai Trading Co., Ltd |
| Boric acid borax solution (1:1) | BH_3_O_3_, B_4_H_20_Na_2_O_17_ | 10043-35-3, 1303-96-4 | ultrapure water | 8 | Tianjin Solomon Biotechnology Co., Ltd |
| Miconazole Nitrate | C_18_H_15_C_l4_N_3_O_4_ | 75319-48-1 | ultrapure water | 5 | Tianjin Huaxun Medical Technology Co., Ltd |
| Cinnamaldehyde | C_9_H_8_O | 104-55-2 | mixture of 1% dimethyl sulfate and 0.1% Tween-80 | 3 | Tianjin Solomon Biotechnology Co., Ltd |
| Glucosinolate crude extracts | None | None | methanol | 7 | Nankai University |
| 5,5-dimethyl-1,3-cyclohexanedione | C_8_H_12_O_2_ | 126-81-8 | DMSO | 6 | Tianjin Laibo Technology Co., Ltd |

Note: Glucosinolate crude extracts were extracted from cauliflower and provided by Professor Chunguo Wang of Nankai University, the molecular formula has not been identified. K100 is a commercial biocide, Euxyl®K100, its main ingredient is 2-methyl-4-isothiazolin-3-one.

**Supplementary Table 2**. Information of fragments of pottery figurines used in the simulation experiment.

| Number | Cultural relic number | Date of excavation | Reagent used for spraying |
| --- | --- | --- | --- |
| A1 | HS M83:8-4 | 2020.11.30 | solvent of cinnamaldehyde |
| A2 | HS M83:8-3 | 2020.11.30 | solvent of cinnamaldehyde |
| A3 | HS M83:8-1 | 2020.11.30 | solvent of cinnamaldehyde |
| B1 | HS M40:5-1 | 2020.10.20 | 50 mg/mL cinnamaldehyde |
| B2 | HS M83:8-7 | 2020.11.30 | 50 mg/mL cinnamaldehyde |
| B3 | HS M150:8-2 | 2021.3.25 | 50 mg/mL cinnamaldehyde |
| C1 | HS M150:8-4 | 2021.3.25 | ddH_2_O |
| C2 | HS M230:4-1 | 2020.7.13 | ddH_2_O |
| C3 | HS M150:6-4 | 2021.3.25 | ddH_2_O |
| D1 | HS M148:3-2 | 2020.12.18 | 0.5% K100 |
| D2 | HS M83:8-5 | 2020.11.30 | 0.5% K100 |
| D3 | HS M150:8-1 | 2021.3.25 | 0.5% K100 |


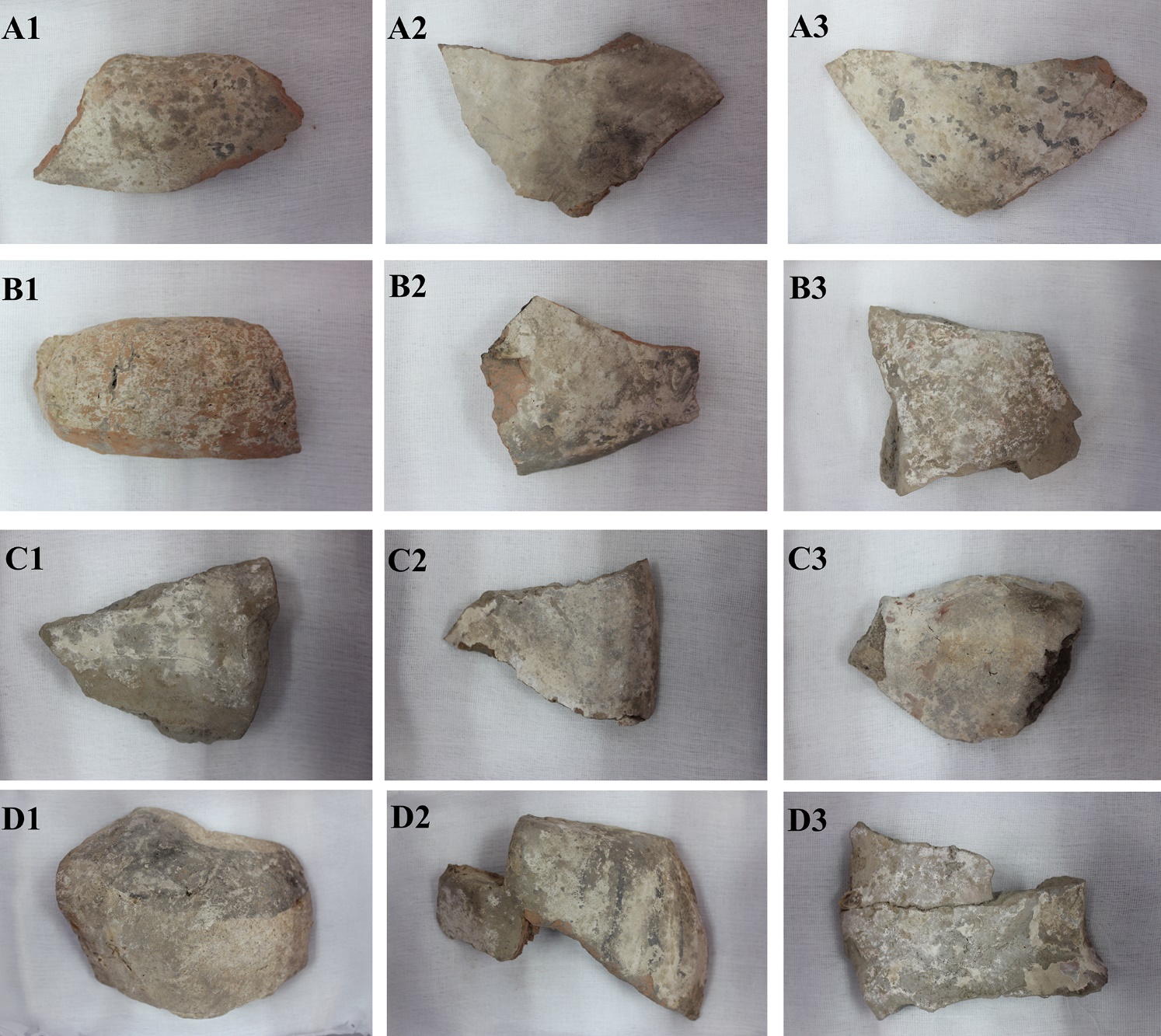


**Supplementary Figure 3**. The original state of pottery figurine fragments used in the simulation experiment. The number is to prepare for later simulation experiment.
